# Antibody Profiling of COVID-19 Patients in an Urban Low-Incidence Region in Northern Germany

**DOI:** 10.3389/fpubh.2020.570543

**Published:** 2020-09-22

**Authors:** Werner Solbach, Julia Schiffner, Insa Backhaus, David Burger, Ralf Staiger, Bettina Tiemer, Andreas Bobrowski, Timothy Hutchings, Alexander Mischnik

**Affiliations:** ^1^Center for Infection and Inflammation Research, University of Lübeck, Lübeck, Germany; ^2^Health Protection Authority, Lübeck, Germany; ^3^Department of Public Health and Infectious Diseases, Sapienza University of Rome, Rome, Italy; ^4^Municipal Statistics Department, Lübeck, Germany; ^5^Gemeinschaftspraxis Huextertor, Lübeck, Germany; ^6^Laboraerztliche Gemeinschaftspraxis Lübeck, Lübeck, Germany

**Keywords:** COVID-19, SARS-CoV-2, immunoglobulin, IgG, IgA, seroprevalence, herd immunity

## Abstract

A vast majority of COVID-19 cases present with mild or moderate symptoms. The study region is in an urban and well-defined environment in a low-incidence region in Northern Germany. In the present study, we explored the dynamics of the antibody response with respect to onset, level and duration in patients with confirmed SARS-CoV-2 infection. Anti-SARS-CoV-2 IgG and IgA were detected by automated enzyme-linked immunosorbent assay (ELISA) of SARS-CoV-2 infected patients monitored by the Health Protection Authority. This explorative monocentric study shows IgA and IgG antibody profiles from 118 patients with self-reported mild to moderate, or no COVID-19 related symptoms after laboratory-confirmed infection with SARS-CoV-2. We found that 21.7% and 18.1% of patients were seronegative for IgA or IgG, respectively. Clinically, most of the seronegative patients showed no to only moderate symptoms. With regard to antibody profiling 82% of all patients developed sustainable antibodies (IgG) and 78% (IgA) 3 weeks or later after the infection. Our data indicate that antibody-positivity is a useful indicator of a previous SARS-CoV-2 infection. Negative antibodies do not rule out SARS-CoV-2 infection. Future studies are needed to determine the functionality of the antibodies in terms of neutralization capacity leading to personal protection and prevention ability to transmit the virus as well as to protect after vaccination.

## Introduction

The novel severe acute respiratory syndrome coronavirus (SARS-CoV-2) causes a respiratory disease, known as COVID-19 (1). On December 31, 2019, Chinese officials reported a cluster of cases of pneumonia in Wuhan, China. The infection was quickly qualified as epidemic (2). As of January 30, 2020, it was announced a public health emergency of international concern. As of March 11, 2020 WHO officially declared the epidemic a pandemic (World Health Organization, Director-General's Opening Remarks at the MediaBriefing on COVID-19 - 11 March 2020). Early reports from China and Italy indicated that SARS-CoV-2 causes illness of varying degrees (3). A vast majority of COVID-19 cases present with mild or moderate symptoms ranging from fatigue, sore throat, cough and fever to a more severe disease course including acute respiratory distress syndrome and septic shock (4, 5). The infection can spread easily as the virus is able to transmit during the presymptomatic or asymptomatic phase of infection (6, 7). In Germany, the first COVID-19 case was detected on January 27, 2020 and spread rapidly around the country (8, 9). On February 28, 2020, Germany's national Public Health Institute (Robert Koch Institute [RKI]) rated the risk of the COVID-19 pandemic for the population in Germany as “low to moderate,” which was then revised to “high” (March 17, 2020) and to “very high” for risk groups (March 26,2020)^1^.

The core basis for the management of the outbreak is the early detection of SARS-CoV-2 in respiratory specimens (nasopharyngeal swabs) from patients presenting with clinical signs such as fever, dry cough or shortness of breath or in asymptomatic persons with close contact to a laboratory confirmed COVID-19 case. People who have a cumulative face-to-face contact with a confirmed case for ≥15 min, direct contact with secretions or body fluids of a patient with confirmed COVID-19 disease, or, in the case of health-care workers, work within 2 m of a patient with confirmed COVID-19 disease without personal protective equipment are at high risk for infection (10). The gold standard for SARS-CoV-2-detection is a specific polymerase chain reaction (PCR) testing from a nasopharyngeal swab, sputum, or broncoalveolar lavage (11). Recently, commercial assays for serological analysis of specific COVID-19 antibodies became available (12, 13). From a public health perspective it is an easy to establish and cost effective laboratory-based screening strategy that may assist in rapid case detection and surveillance and ultimately in a better understanding of this epidemic (10). Since there is no specific medical treatment or a vaccine available at present, it is crucial that sufficient herd immunity will develop in the population to interrupt uncontrollable transmission of the virus. Like in other coronaviruses, it is likely that neutralizing antibodies are central to the development of herd immunity to SARS-CoV-2. Therefore, insight into the development of immunity is pertinent for future guidance of preventive measures. In addition, antibody levels may give information on whether patients with COVID-19 infection are immune to re-infection. However, given that SARS-CoV-2 is a newly emerging virus, the antibody response remains largely unknown.

At present, different investigations are ongoing to get insight in seroprevalence of COVID-19 infection in Germany and Europe. Many researchers report from hot spot areas in Europe (14, 15) or regions of high prevalence in Germany (16). The extent, duration and the protective function of the antibody response are not clear. The infection rate in northern Germany has been milder than in other parts of the country. Due to rigorous containment measures and early contact tracing, the city of Luebeck had an incidence rate that was lower than the average incidence in Germany.

In the present study, we explored the dynamics of the antibody response with respect to onset, level and duration in patients with confirmed SARS-CoV-2 infection in this low incidence region. Most of the study patients were outpatients with either mild, moderate or even without symptoms. The disease severity of all patients was manageable by doctors in general practice (GP) or as outpatients in the local clinics. The precise knowledge of the disease severity allowed us to attempt the clinical validation of the antibody development.

## Materials and Methods

### Study Population and Participant Recruitment

**Figure 1:**
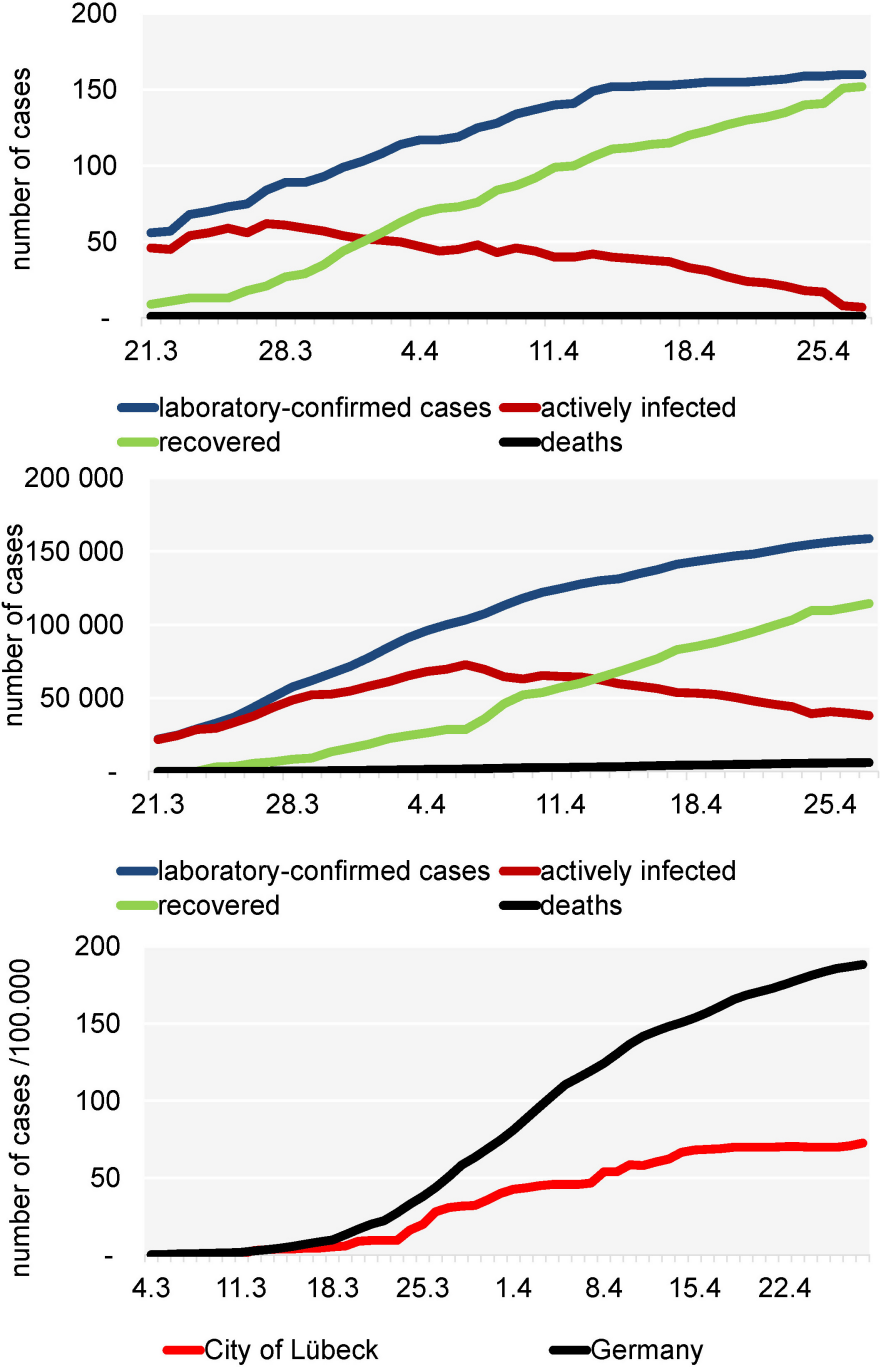
Development of the COVID-19 pandemic in the City of Lübeck **(above)**, in Germany **(middle)** and incidence/100,000 inhabitants in the city of Lübeck **(below)**.

**Figure 2:**
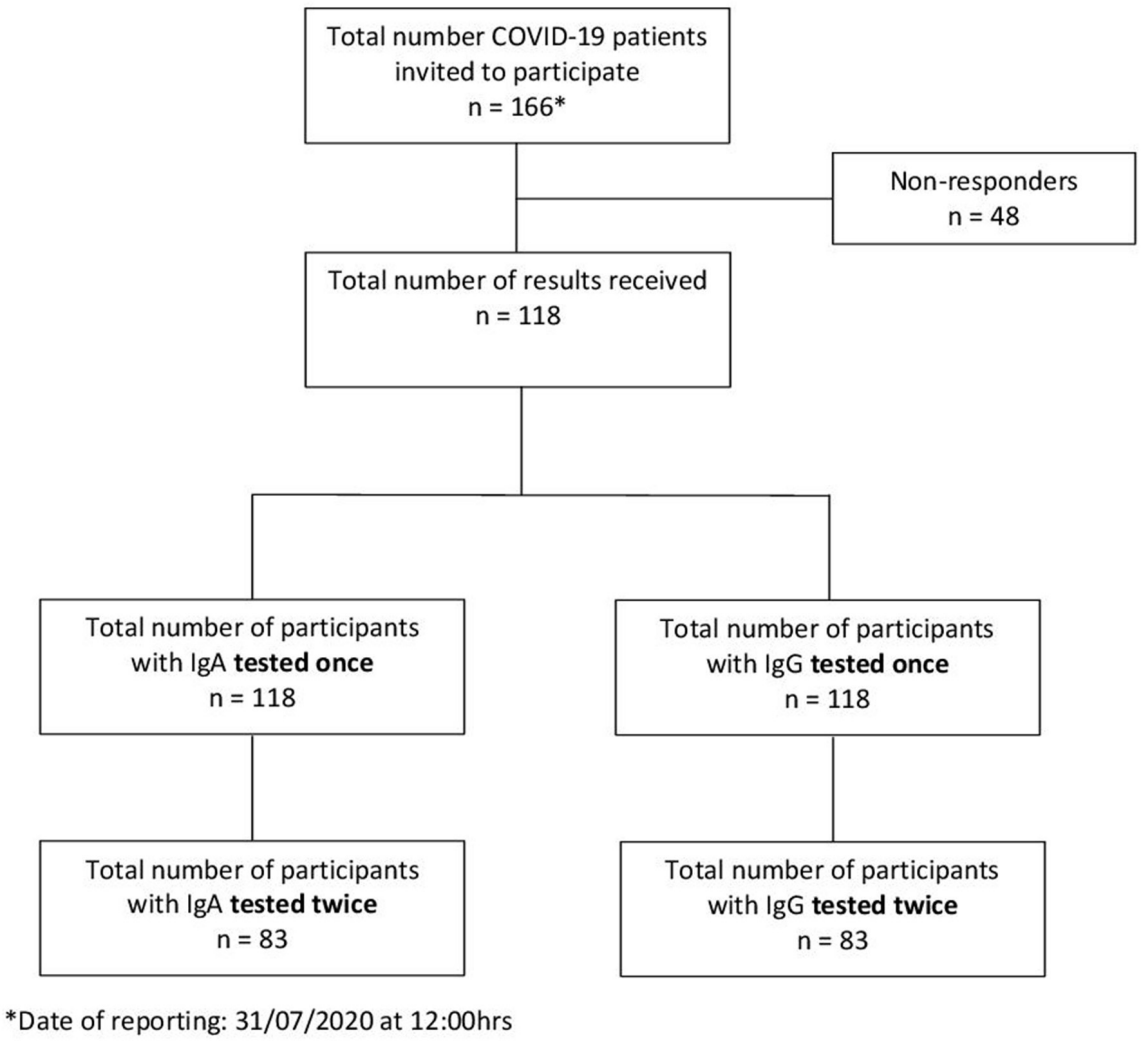
Flowchart of participant enrollment.

The city of Luebeck, with a population of 220,238 inhabitants, is situated in Northern Germany. The first two laboratory-confirmed SARS-CoV-2 cases were detected on February 29, 2020. The epidemic grew to 166 cases from which 151 recovered and one person (79 years) died (as of July 31, 2020) (Figure 1 above). A total of 166 confirmed COVID-19 patients who fulfilled the RKI definition^2^ were detected by the local health authority between February 27, and July 31, 2020 (Figure 2) and were enrolled in the study. All enrolled cases were confirmed to be SARS-CoV-2 infected by use of a standard polymerase chain reaction (PCR) assay on throat swab samples from the respiratory tract taken by the local health authority. According to the guidelines, patients were quarantined routinely for at least 14 days from the onset of symptoms or from laboratory testing, respectively, in the absence of symptoms. Of these 166 index cases, 118 gave their written informed consent to participate (Figure 2). For children under the age of 18 years parents or other legal guardians provided “informed permission/consent” for study participation. For all of the enrolled patients, the date of symptom onset, disease severity (e.g., hospitalization) and demographic information were obtained from clinical records available to the local health authority and through a self-reported questionnaire.

### Test Procedures

#### Detection of SARS-CoV-2

Nasopharyngeal swabs were taken from suspected COVID-19 cases by trained professionals either in a general practice (GP) or in a “drive-in” swab center run by the Health Protection Authority. Swabs were stored in stabilization media and processed immediately within 4 h, following DIN EN ISO 17025 und 15189 quality criteria, in the “Laboraerztliche Gemeinschaftspraxis Luebeck,” which is located in the immediate vicinity of the “drive-in” swab center. SARS-CoV-2 RNA was detected qualitatively by using an automated one step real-time RT-PCR (RIDA®GENE SARS-CoV-2 RUO Test; R-Biopharm AG, Darmstadt, Germany; E-gene amplification) run on a RIDA®CYCLER according to the manufacturer's instruction.

#### Detection of IgG and IgA Against SARS-CoV-2

Anti-SARS-CoV-2 IgG and IgA were detected by automated enzyme-linked immunosorbent assay - ELISA (product EI 2606-9601 G or A; EUROIMMUN; https://www.euroimmun.com) according to the manufacturer's instructions. The ELISA plates are coated with recombinant protein expressed S1 domain glycoprotein as antigen of the SARS-CoV-2. According to the latest data sheet (April 2020), the specificity for IgG testing is reported to be 99.1% for IgG and 88.5% for IgA, respectively. The data sheet reports cross-reactivities with SARS-CoV-1, but not with MERS-CoV, HCoV-229E, HCoV-NL63, HCoVHKU-1, or HCoV-OC43 virus. Thus, possible cross-reactivities are, at most, of marginal importance for this study, since very little, if any, SARS-CoV-1 infection is to be expected. The optical density (OD) was detected at 450 nm. A ratio of the OD of each sample to the reading of the calibrator, included in the kit, was automatically calculated according to the formula: OD ratio = OD of serum sample/OD of calibrator. According to the manufacturer, a ratio below 0.8 was evaluated as negative, 0.8 – <1.1 as borderline and >-1.1 as positive.

### Statistical Analysis

Baseline and demographic characteristics of the patients were summarized by standard descriptive statistics. In order to investigate the seroconversion, the data from 118 sera samples were divided into six time windows of collection after symptom onset:

- 0–7 days
- 8–14 days
- 15–21 days
- 22–28 days
- 29–36 days
- 37 and more days

Categorical variables are expressed as numbers and percentages. Since data was not normally distributed, non-parametric tests were chosen. Spearman's correlation was conducted to assess the correlation between age and disease severity and antibody load (IgA and IgG load). A *p* < 0.05 was considered statistically significant. Statistical analyses were conducted using SPSS version 26.0.

## Results

### Local and National Development of the COVID-19 Pandemic

Figure 1 above shows that from March 21 the number of active cases increased in a linear fashion and reached a plateau-like curve after April 16. From April 1 on, the number of recovered patients always exceeded the active cases. Until July 30, in total 166 COVID-19 laboratory-confirmed cases have been reported to the local health authority of Luebeck. One patient died due to COVID-19. For Germany, until April 30th, in total 161.539 COVID-19 laboratory-confirmed cases and 6.467 deaths due to COVID-19 were reported (Figure 1 middle). After March 16, the local incidence rate always was lower than the national incidence rate (Figure 1 below) and since April 15 it was relatively stable at 68.1–72.6/100,000, while the incidence in the rest of Germany was steadily increasing (~189/100,000) as of April 27. A total of 118 patients with a confirmed diagnosis of COVID-19 were included in this study. All patients were positive for SARS-CoV-2 according to PCR testing of nasopharyngeal swabs.

### Sample Characteristics

Clinical and demographic characteristics are reported in Table 1. Among all notified cases, 7 (5.9%) were children or adolescents aged 10–19 years, 88 (72.9%) persons were aged 20–59 years, 23 (19.5%) persons were aged 60–79 years, 2 (1.7%) persons were aged 80 and older (Table 1). Sixty-seven patients (56.8%) were female and 51 patients (43.2%) were male (Table 1).

**Table 1:** Clinical and demographic characteristics of the patients included (*n* = 118).

**Characteristics**	***N* (%)**
**Gender**	
Female	67 (56.8)
Male	51 (43.2)
**Age groups**	
10–19	7 (5.9)
20–29	22 (18.6)
30–39	18 (15.3)
40–49	14 (10.2)
50–59	34 (28.8)
60–69	17 (14.4)
70–79	6 (5.1)
80 and older	2 (1.7)
**Disturbance of smell and/or taste (data available for 105 patients)**	
Yes	64 (61.0)
No	41 (39.0)
**Disease category (data available for 105 patients)**	
1. No symptoms	6 (5.7)
2. Feeling of illness, but temperature <38°C	45 (42.9)
3. General weakness, dry cough, temperature >38°C (influenza like illness)	45 (42.9)
4. As in 3 plus shortness of breath, signs of pneumonia	6 (5.7)
5. As in 4, hospital treatment required	3 (2.9)

#### Disturbance of Smell and Taste

More than half (61%) of the patients self-reported slight to massive decrease in one or both senses (i.e., taste or smell). Although we did not quantify, they reported a duration between one and up to 4 weeks after recovery from the acute illness.

#### Disease Severity

Patients exhibiting one or more of the following conditions were classified as having severe COVID-19: Fever above 38°C and signs of pneumonia accompanied by shortness of breath (i.e., category 4); Hospitalization (respiratory failure requiring mechanical ventilation and ICU care) (i.e., category 5).

Patients not meeting the above criteria, but exhibiting one or more of the following conditions classified as having mild COVID-19: feeling of illness, but temperature <38°C (i.e., category 2); General weakness, dry cough, temperature >38°C (influenza like illness) (i.e., category 3).

Among the 118 patients, ~6% of showed no symptoms; 86% had mild COVID (category 2 and 3) and 9% were severe cases (category 4 and 5) (Table 1). The main symptom was general weakness with or without headache or body ache, but no fever. We could not find a significant correlation with age and disease severity (*p* > 0.05) and neither an association between gender and disease severity (*p* > 0.05).

### Antibody Profiling

As of July 31, 2020, IgA and IgG antibody results from 118 participants were available. A total of 83 patients were tested twice to monitor the course of antibody development (Figure 2).

Antibody levels for the first and second testing are shown in Table 2. According to the manufacturer's data sheet, an antibody ratio ≥ 1.1 is defined as positive, where as an antibody ratio <0.8 is defined as negative. In the present sample, 21.7 and 18.1% of patients were seronegative for IgA or IgG, respectively (Table 2). Clinically, most of the seronegative patients showed no to only moderate symptoms. Among the six asymptomatic patients, two patients did not develop IgG antibodies (not shown). Twenty nine (24.5%) patients and 34 (28.8%) patients had high IgG and IgA antibody levels above 5 in the first testing, respectively (Table 2). Again, most of them had no to moderate symptoms thus were in category 1 to 3 (not shown).

**Table 2:** IgA and IgG antibody levels against SARS-CoV-2 in COVID-19 patients.

**Antibody levels**	IgA (1)^*^ (*n* = 118)	IgA (2)^*^ (*n* = 83)	IgG (1)^**^ (*n* = 118)	IgG (2)^**^ (*n* = 83)
	***N* (%)**	***N* (%)**	***N* (%)**	***N* (%)**
<0.8	23 (19.5)	18 (21.7)	28 (23.7)	15 (18.1)
0.8–0.9	6 (5.1)	4 (4.8)	7 (5.9)	3 (3.6)
1.0–2.0	21 (17.8)	25 (30.1)	25 (21.2)	18 (21.7)
2.1–5.0	34 (28.8)	22 (26.5)	29 (24.6)	23 (27.7)
5.1–10.0	29 (24.6)	11 (13.3)	26 (22.0)	23 (27.7)
10.1–15.0	2 (1.7)	2 (2.4)	3 (2.6)	1 (1.2)
15.1–20.0	0 (0.0)	0 (0.0)	0 (0.0)	0 (0.0)
20.1–25.0	1 (0.8)	0 (0.0)	0 (0.0)	0 (0.0)
≥25.1	2 (1.7)	1 (1.2)	0 (0.0)	0 (0.0)

* (1) First test

** *(2) Second test*.

**Figure 3:**
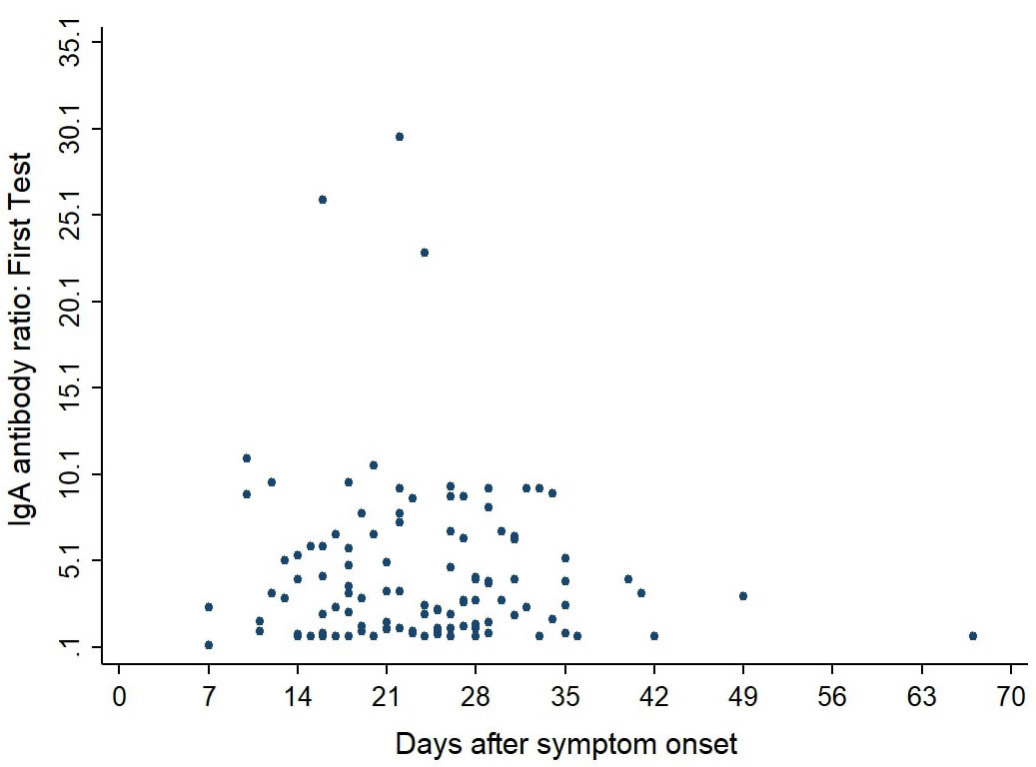
Simple Scatter of IgA Antibodies by days after symptom onset.

**Figure 4:**
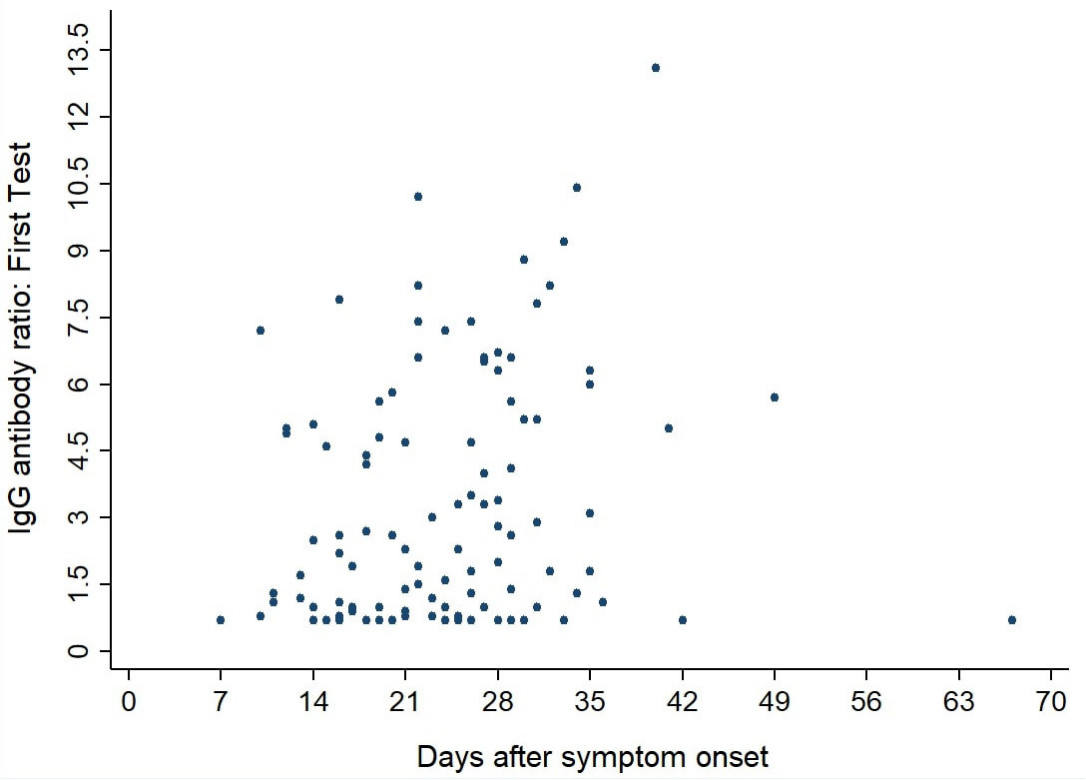
Simple Scatter of IgG Antibodies by days after symptom onset.

**Figure 5:**
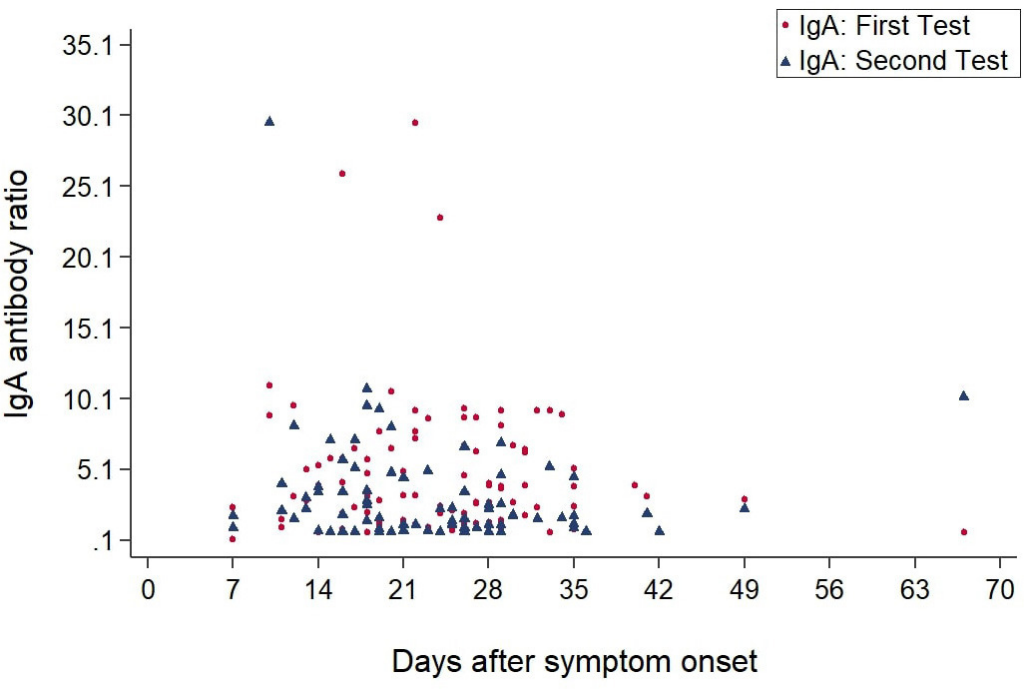
IgA antibody development by days after symptom onset.

**Figure 6:**
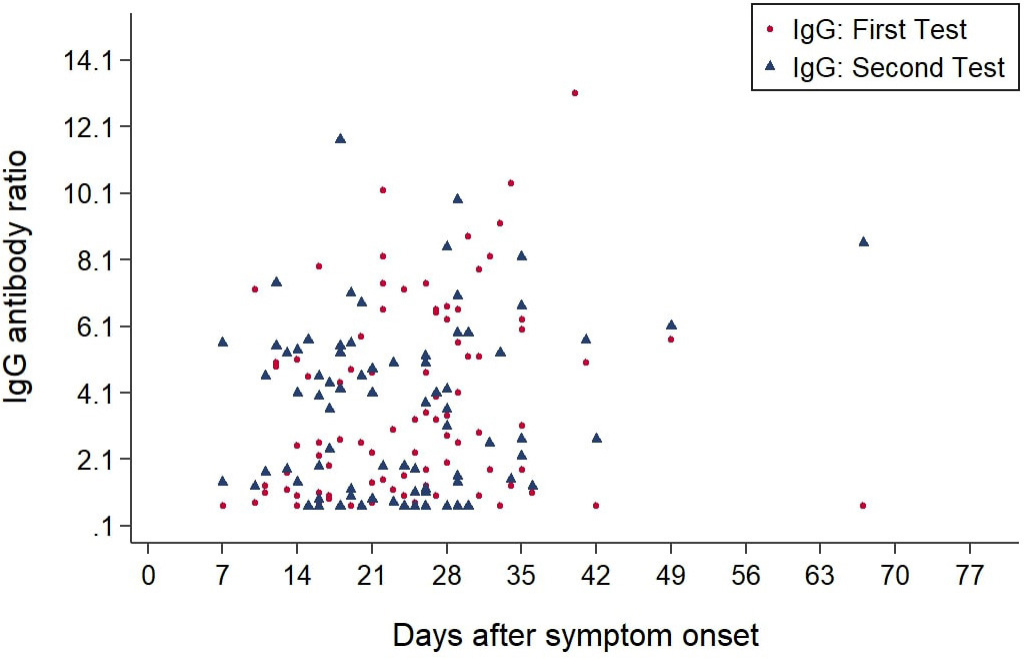
IgG antibody development by days after symptom onset.

Figures 3, 4 show the antibody levels for IgA and IgG, respectively, in relation to symptom onset. Antibodies were analyzed between day 7 and day 67 after symptom onset. As shown in Figure 3 and Table 3, anti-SARS-CoV-2 S-specific IgA and IgG antibodies were not detectable in the very early days of infection (from day 0 to day 6). The first positive signals were detected at day 7 for IgA antibodies. Positive levels for IgG were detected between 8 and 14 days after symptom onset (Figures 3, 4, Table 3). There was a wide inter-individual variation in the antibody levels. Figure 5 indicates that IgA levels remain fairly stable, but increase at around day 50. A similar pattern could be detected for IgG levels, although IgA levels seem to increase over time (Figure 6).

**Table 3:** IgA and IgG detection in positive COVID-19 patients at different periods after disease onset.

**Days after symptom onset**	IgA positive* (ratio ≥1)	IgG positive* (ratio ≥1)
	***N* (%)**	***N* (%)**
0–7	1 (1.1)	0 (0.0)
8–14	10 (11.2)	10 (12.0)
15–21	24 (27.0)	19 (22.9)
22–28	31 (34.8)	30 (36.1)
29–36	20 (22.5)	21 (25.3)
≥37	3 (3.4)	3 (3.7)

*Antibody levels here coded as binary outcome: IgA level ≥1.0 = positive; IgA level <1.0 = negative; IgG level ≥1.0 = positive; IgG level <1.0 = negative*.

## Discussion

Our analysis of SARS-CoV-2 cases provides insight into the development of immune response in patients with light or moderate course of COVID-19 disease or asymptomatic patients.

Our cohort of patients was defined as having had viral mRNA in nasopharyngeal swabs by qualitative RT-PCR. The viral load was not analyzed quantitatively. The severity of diseases was very variable from completely asymptomatic to pneumonia-like symptoms. The severity of the symptoms may be dependent on the viral load at the time of infection. In Germany, the first and only commercially available test kit came into the market as of March 25, 2020. Based on the experience with the SARS-CoV-1 epidemics in 2003, the manufacturer (who also offers an antibody kit detecting SARS-CoV-1) decided to include IgA and IgG. The rationale behind this was, as suggested, that IgA would be much earlier and more specific than IgM and was expected to indicate mucosal immunity.

We analyzed IgA and IgG antibodies recognizing the S1 spike glycoprotein of SARS-CoV-2 in the serum of 118 SARS-CoV-2 PCR-positive patients with mild-to-moderate symptomatic or asymptomatic course in a low COVID-19-incidence region in northern Germany. Overall, more women (56.8%) than men tested positive for SARS-CoV-2, which is in line with overall findings for Germany as a whole (52%)^3^.

With regard to the initial stratification the course of the disease was mild to moderate. Most patients, with a few exceptions, did not require hospital treatment, but most were treated by their general practitioner. Patients were treated by their family doctors. More than half self-reported sudden disturbances of smell and/or taste with varying durations (days to weeks) (17). The extent of olfactory dysfunction did not correlate with age, sex, or severity of the disease (not shown).

The antibody tests specifically interact with S1. It is likely, that other antigens like the viral nuclecapsid (NCP) antigen also induce antibody production. In the analysis of antibody levels, it appeared that, within a period of 50 days after the infection, 95/118 (81%) and 90/118 patients (76%) of the patients developed antibodies for IgA and IgG, respectively, above the threshold ratio of 1.1 at the first testing (Table 2). The level of antibodies, however, did not correlate significantly with age or sex or disease severity. Remarkably, 18% of the patients, with or without symptoms, did not develop IgG antibodies above the cut-off-value (1.1) after two testings. Again, no correlation with sex, age or disease severity could be observed (not shown). Our findings are in line with recent reports by colleagues who reported around 30% IgG-negative patients after SARS-CoV-2-infection (18). Others recently reported that mild disease – like in most of our cases – may stimulate mucosal SARS-CoV-2 secretion and IgG production may be associated mainly with severe cases (19).

The accumulating evidence supports a role for T cells in COVID-19 and probably in the immunological memory that forms following recovery from SARS-CoV-2 infection (20). Most, although not all, patients who are hospitalized seem to mount both CD8+ and CD4+ T cell responses, and evidence points to possible suboptimal (lymphopenia), excessive or otherwise inappropriate T cell responses associated with severe disease. Very rare data are available on T-cell responses from patients with mild to moderate disease like in our cohort.

It remains to be determined, whether S1 or NCP has the better positive or negative predictive value, as far as protection or resistance to re-infection is concerned.

We can only speculate that the antibodies are neutralizing. Therefore, at this time, it will not be possible to testify protection for recovered patients. The severity of symptoms may be dissociated from the antigenicity or presentation of the S1 glycoprotein to the immune system. This assumption would explain the high antibody variability which was not related to expression of clinical symptoms.

Six (5.7%) of the patients did not develop any SARS-CoV-2 specific symptoms. One out of these did not have detectable antibodies within 50 days. Thus, a negative antibody test does not rule out infection. The infectious inoculum possibly was not sufficiently high to induce disease and a subsequent immune reaction. Thus, both, a combination of SARS-CoV-2 PCR and a specific serological test are required to rule out infection (21).

One of the asymptomatic patients had an IgG ratio > 5. Although unlikely, it cannot be excluded entirely, that the patient was infected some weeks before the virus was detected and, thus, would have been protected in the observation period of this study. In any case, the data suggest that activation of the humoral immunity might require less virus than activation of symptomatic disease processes. Further studies are needed to define the respective minimal viral loads.

When we investigated the antibody levels in relation to time, it was obvious that there was a great diversity for both IgA and IgG. For IgA we found one positive value (IgA ratio ≥ 1.1) starting at day 7 after symptom onset (Table 3). For IgG, positive values (IgG ratio ≥ 1.1) were detected roughly 8–14 days after disease onset (Table 3). This is in line with other research that found that among most laboratory-confirmed COVID-19 cases, antibodies start to be detectable around 5–14 days after onset of symptoms (22–25). The test systems used were different. It remains to be seen, which antigen (nucleocapsid, spike glycoprotein) is the best to detect relevant antibodies. We could not find a correlation between antibody levels and age (*p* > 0.05).

Based on these findings, it is clear, that antibody diagnosis is a significant pillar to identify COVID-19 -positive patients in addition to SARS-CoV-2 PCR. It also will be indispensable for management of the pandemic. It is likely, that antibodies directed against the S1 glycoprotein are neutralizing (16).

In the cited study, most of the patients, but not all, with an IgG ratio above 2, had positive neutralization titres. They used the same test system as we did in our study. Thus, our data add information, but do not prove protection. For the individual patient, however, it cannot be answered at present, which antibody level will be protective and whether antibody-positive individuals are able to transmit the disease. Further studies are needed to answer this question, which is of utmost importance e.g., for health care workers.

There are some limitations in our study. Cross-reactivity could possibly be a limitation of immunoassays. On the one hand, our test was validated with a sensitivity of 89–100% and a specificity of 87.5–95.5% for IgA and 83.5–97.5% for IgG and a recent study has demonstrated negligible cross-reactivity from other human coronavirus NL63 to SARS-CoV-2 (12, 26). Our study does not give information on protective antibody functions with regard to resistance to re-infection and reduction of transmissibility of the virus. The results on the neutralization capacity are not present till now. Based on the development of IgG antibody dynamics, however, it might be reasonable to assume that ratios beyond 2 might confer protection. Nonetheless, this study provides valuable information regarding the seroconversion response, especially for IgA and IgG antibody development.

## Conclusion

In the present study we detected that ~2 weeks after infection the majority of symptomatic patients develop IgA and IgG antibodies. Our findings demonstrate that antibody tests have important diagnostic value in addition to RNA tests. In patients with viral RNA detection by PCR, but in the absence of symptoms, significant antibody levels were not detectable in a relevant proportion. This finding raises the question of false-positive PCR results that has to be investigated in further studies. Our data indicate, however, that antibody-positivity is a useful indicator of a previous SARS-CoV-2 infection. Negative antibodies cannot rule out SARS-CoV-2 infection. A number of questions still have to be answered. For the clinic, the determination of the neutralizing capacity of the antibodies in plasma therapy regimes will be of utmost relevance. On a population level, the protective effect for re-infections needs to be determined.

## Data Availability Statement

The raw data supporting the conclusions of this article will be made available by the authors, without undue reservation.

## Ethics Statement

The studies involving human participants were reviewed and approved by Ethics committee University of Lübeck. Written informed consent to participate in this study was provided by the participants' legal guardian/next of kin.

## Author Contributions

WS and JS designed the study and analyzed the data. IB, DB, and TH performed statistical analysis and database management. RS delivered clinical information and serum specimens. BT and AB performed laboratory tests (PCR and antibody tests). WS, JS, and AM wrote the manuscript. All authors reviewed the manuscript and gave their consent.

## Conflict of Interest

The authors declare that the research was conducted in the absence of any commercial or financial relationships that could be construed as a potential conflict of interest.

## Footnotes

^1^https://www.rki.de/DE/Content/InfAZ/N/Neuartiges_Coronavirus/Situationsberichte/2020-03-26-de.pdf?__blob=publicationFile.

^2^https://www.rki.de/DE/Content/InfAZ/N/Neuartiges_Coronavirus/Falldefinition.pdf?__blob=publicationFile

^3^https://www.rki.de/DE/Content/InfAZ/N/Neuartiges_Coronavirus/Situationsberichte/2020-05-05-de.pdf?__blob=publicationFile

## Abbreviations

COVID-19: coronavirus disease 19
ELISA: enzyme-linked immunosorbent assay
IgG/A: immunoglobulin G/A
rtPCR: real-time polymerase chain reaction
SARS-CoV-2: Severe acute respiratory syndrome coronavirus 2.
